# Insulin resistance phenotype is associated with vascular risk phenotype at the end of the second decade of life: a population-based study

**DOI:** 10.1186/s12933-022-01724-0

**Published:** 2022-12-19

**Authors:** Janaina Maiana Abreu Barbosa, Antônio Augusto Moura da Silva, Rosangela Fernandes Lucena Batista, Bernadete Jorge Leal Salgado, Joelma Ximenes Prado Teixeira Nascimento, Vanda Maria Ferreira Simões, Maria Jacqueline Silva Ribeiro, Marco Antonio Barbieri, Alexandre Archanjo Ferraro, Cecilia Claudia Costa Ribeiro

**Affiliations:** 1 Graduate Program in Public Health, Public Health Department, Federal University of Maranhão, Rua Barão de Itapary, 155 - Centro, São Luís, MA 65020-070 Brazil; 2 Department of Physiological Sciences, Federal University of Maranhão, São Luís, Brazil; 3 Department of Health Sciences, Federal University of Maranhão, São Luís, Brazil; 4 Faculty of Medicine of Ribeirão Preto, Department of Childcare and Pediatrics, University of São Paulo, São Paulo, Brazil; 5 Department of Pediatrics, Faculty of Medicine, University of São Paulo , São Paulo, Brazil

**Keywords:** Adolescents, Blood pressure, VLDL-Cholesterol, Insulin resistance, Pulse wave speed

## Abstract

We hypothesize that early events of diabetes and cardiovascular disease continuums would be ongoing and associated in adolescents. We investigated the association between the *Insulin Resistance Phenotype* and the *Vascular Risk Phenotype* at the end of the second decade of life and indirect pathways from social vulnerability, alcohol consumption, and body fat mass. It is a population-based study in the RPS cohort of 18–19 years (n = 2,515), São Luís, Brazil. The theoretical model analyzed the association between *Insulin Resistance Phenotype* and *Vascular Risk Phenotype* by sex, using structural equation modeling (SEM). The *Insulin Resistance Phenotype* was a latent variable deduced from the correlations of Triglyceride to HDL ratio, Triglyceride Glycemic index, and VLDL; the Vascular Risk Phenotype was deduced from Systolic Blood Pressure, Diastolic Blood Pressure, and Pulse Wave Velocity. The *Insulin Resistance Phenotype* was directly associated with the *Vascular Risk Phenotype* in males (standardized coefficient SC = 0.183; p < 0.001) and females (SC = 0.152; p < 0.001). The *Insulin Resistance Phenotype* was an indirect pathway in the association of alcohol consumption and higher values of fat mass index with the *Vascular Risk Phenotype*. VLDL presented the highest factor loading, appearing as a marker of insulin resistance linked to cardiovascular risk in young people. Lower values of socioeconomic status, harmful use of alcohol, and high body fat values were also associated with higher values of the two phenotypes. The association of the *Insulin Resistance Phenotype* with the *Vascular Risk Phenotype* suggests common pathophysiological mechanisms present in early events in the continuums of diabetes and cardiovascular disease in adolescence.

## Introduction

Cardiovascular severe events such as coronary artery disease, heart failure, and myocardial infarction known as Major Adverse Cardiac Event (MACE) manifest in adults [1]. Decades earlier, in the MACE latency period, the cardiovascular continuum is already ongoing, a chain of earlier pathophysiological events involving oxidative stress, inflammatory responses, endothelial dysfunction and atherosclerosis [2].

Type 2 diabetes also has the continuum characteristic, involving oxidative stress and inflammatory response, which progressively cause changes in insulin signaling pathways, changes in glucose transport and pancreatic beta-cell dysfunction until the clinical diagnosis of the disease [3]. Type 2 diabetes is a recognized risk factor for Cardiovascular Diseases (CVD) [4].

However, even before hyperglycemia installation, excess fatty acids in the pancreas, liver and heart increase insulin secretion and hepatic glucose production, resulting in diastolic dysfunction [5]. In the liver, the release of free fatty acids results in hyperinsulinemia and hypertriglyceridemia by de novo *lipogenesis* [6] and Very-Low-Density Lipoprotein (VLDL) secretion [7]. The increase in triglycerides also results in inflammation and endothelial dysfunction, turning into the ideal environment for atherogenesis, even before type 2 diabetes installation [8].

Thus, dyslipidemia and visceral obesity seem implicated in the genesis of diabetes and CVD [9], with involvement of vascular inflammation and early endothelial dysfunction [10]. We hypothesize that early events of diabetes and cardiovascular continuums would be ongoing and already associated in adolescence, a sensitive period for human development.

We propose the latent variable *Insulin Resistance Phenotype* representing early Type 2 Diabetes continuum events. This *Phenotype* was deduced from the shared variance of the indicators Triglyceride to HDL ratio (TG/HDL), Triglyceride Glycemic Index (TyG) and VLDL levels. None of these isolated indicators measure the *Insulin Resistance Phenotype* well; however, using them together as a continuous latent variable may reduce the error magnitude of measuring insulin resistance in adolescents. At the same time, the *Vascular Risk Phenotype* represents the shared variance between Systolic Blood Pressure (SBP), Diastolic Blood Pressure (DBP) and Carotid-Femoral Pulse Wave Velocity (PWV) to assess incipient vascular alterations in young people [11].

In this study, we investigated the association between the *Insulin Resistance Phenotype* and the *Vascular Risk Phenotype* at the end of the second decade of life, using a model that considered the complexity of this relationship and adjustments for social determinants, alcohol consumption and body fat mass in young people.

## Methods

### Study design

A population-based study was nested within the RPS cohort of 18–19 years (n = 2,515), São Luís, Brazil, from January to November 2016.

**Fig. 1:**
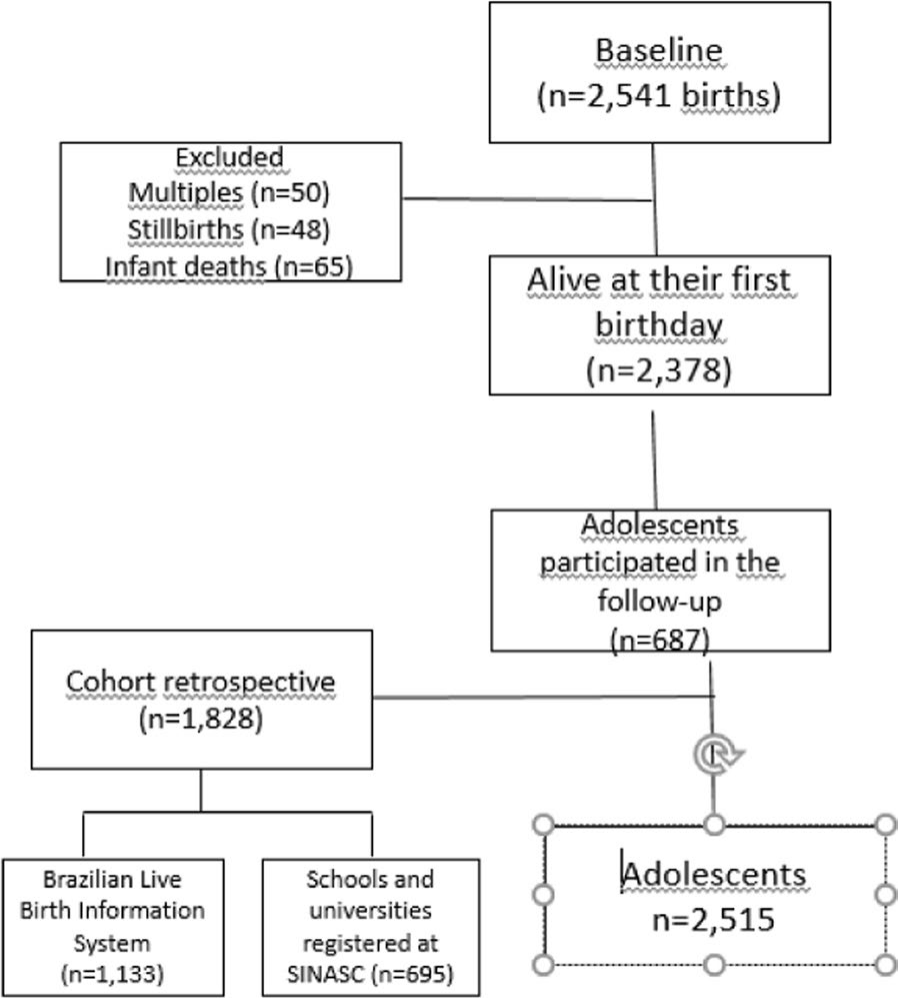
Flowchart of participants. The RPS Cohort follow-up at 18–19 years old (n = 2,515), São Luís, Brazil

All individuals who participated at the beginning of the original cohort were invited to participate in the second follow-up (n = 687). The cohort follow-up also included other individuals born in São Luís, Brazil, in 1997 by drawing from the Brazilian Live Birth Information System (SINASC) (n = 1,133) to increase the sample's power and prevent future losses (retrospective cohort). The second stage included volunteers identified in schools and universities registered at SINASC and born in 1997 (n = 695). Thus, the sample of this study consisted of 2,515 adolescents (Fig. 1).

### Data collection

We collected information from the adolescents: sex, education of the adolescent and the head of household, socioeconomic class, monthly family income, consumption of alcoholic beverages, fat mass index, TG/HDL ratio, TyG index, VLDL levels, SBP, DBP and PWV.

The following indicators were included in the latent variable socioeconomic status: (1) Household head´s education (elementary, secondary and higher education), (2) Adolescent´s education (elementary, secondary and higher education); (3) Socioeconomic class (A, B, C, D-E) using the CCEB criteria of the Brazilian Economic Classification, in which Class A is the wealthiest/most educated; and (4) Monthly household income based on the Brazilian national minimum wage in force in 2016 (USD 270.76). Alcohol consumption was assessed using the Alcohol Use Disorders Identification Test (AUDIT) questionnaire from the World Health Organization and classified as low and high risk.

Fat mass was calculated by dual-energy x-ray absorptiometry (DEXA), and height was measured with a stadiometer (Altura Exata^®^). The fat mass index was used as a body fat parameter, corresponding to the fat mass (kg) divided by the square of the body height (m^2^) and used as a continuous variable.

For blood collection, 40 Ml was obtained from the cubital vein before the snack was served to the adolescents who were fasting for at least 2 hrs to assess triglycerides (TG) (mg/dL), High-Density Lipoprotein (HDL) (mg/dL), blood glucose (mg/dL) and VLDL (mg/dL) contents. These markers were analyzed using the Sysmex XE-2100^®^ hematology analyzer.

The TG/HDL ratio has been used as a metabolic marker of insulin resistance [12]. The TyG index is a marker of insulin resistance more accessible to clinical practice than the insulin resistance index (HOMA-IR). TyG was calculated by multiplying blood glucose by triglycerides, as in formula: Ln [Triglyceride (mg/dL) x fasting glucose (mg/dL)/2], where Ln is the Naperian logarithm [13]. The VLDL concentration, which has high concentrations of triglycerides, is also used as a marker of insulin resistance in adolescents [14]. All the indicators of insulin resistance were used as continuous variables.

Blood pressure was measured with the oscillometric method using the OMRON HEM-742INT^®^ automated cuff device (Omron, São Paulo, Brazil). Three measurements were taken after at least 5 min at rest with a 1 min interval, in a sitting position, with the dominant arm supported at the heart level. The mean of three values represented the adolescent´s blood pressure in the analysis.

### Latent variables

This study used as latent variables:

1. *Family Socioeconomic Status* deduced from the shared variance of the indicators household head´s education, education of the adolescent, socioeconomic class according to CEB criteria and family monthly income;
2. *Insulin Resistance Phenotype* deduced from the shared variance of TG-HDL ratio, TyG index and VLDL concentration;
3. *Vascular Risk Phenotype* deduced from the shared variance of SBP, DBP and PWV.

### Proposed theoretical model

**Fig. 2:**
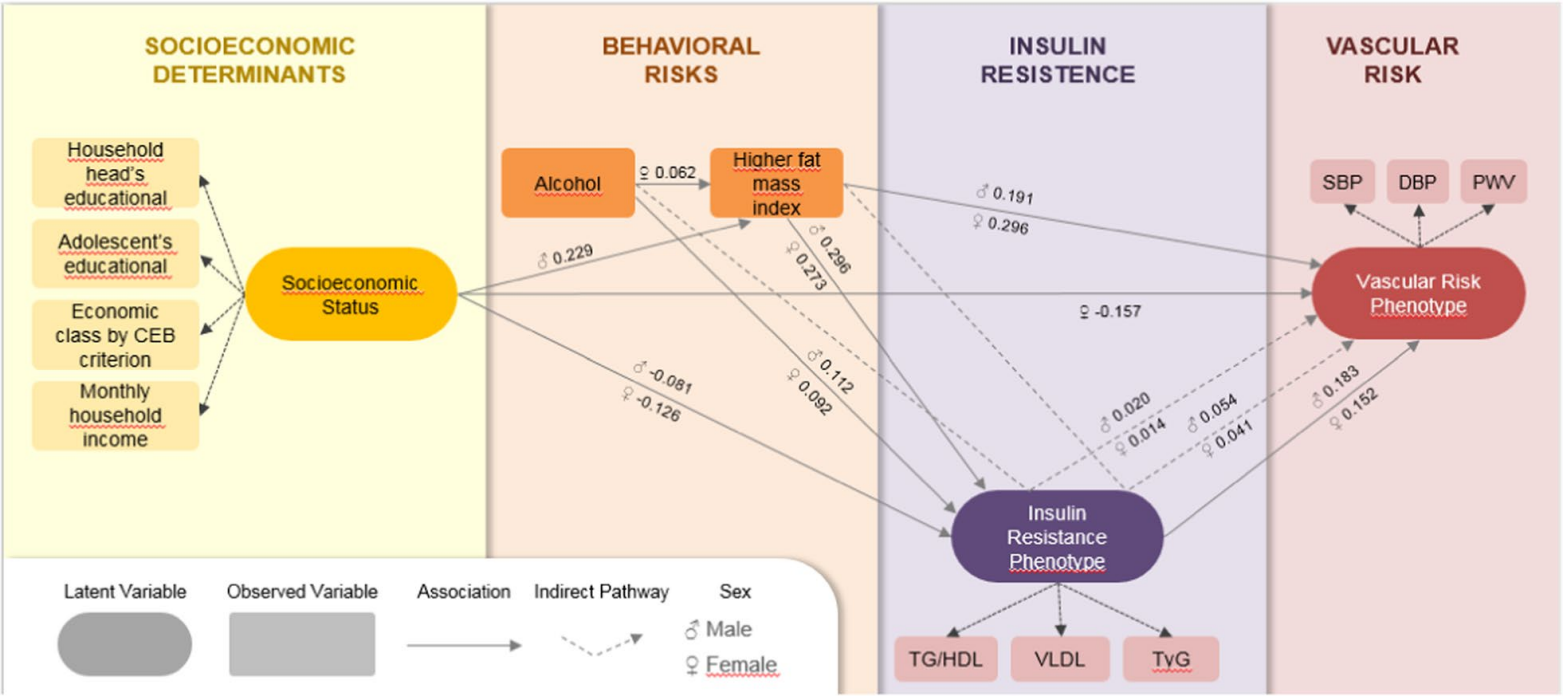
Proposed theoretical model and estimates of the association between the Insulin Resistance Phenotype with the Vascular Risk Phenotype in adolescents. São Luís, Brazil (2022)

The latent *Family Socioeconomic Status* was considered the most distal determinant and would explain all other variables in the model. Harmful use of alcohol would be associated with fat mass index, *Insulin Resistance Phenotype* and *Vascular Risk Phenotype*. The fat mass index would be associated with the *Insulin Resistance Phenotype* and the *Vascular Risk Phenotype*. The *Insulin Resistance Phenotype* would be related to the *Vascular Risk Phenotype *(Fig. 2).

### Statistical analysis

Descriptive data were analyzed using the STATA^®^ statistical software, version 16.0. Absolute and relative frequencies described categorical variables. Females and males were tested in separate models, using Structural Equation Modeling (SEM). SEM performs confirmatory factor analysis and estimates a series of multiple regression equations. The main function is the specification and estimation of models of linear relationships between variables observed (effect indicators) or latent (constructed). Latent variables are constructs derived from the combination of observed ones, extracting the common variance shared by these effect indicators. A latent variable estimates effect free from bias originating from measurement errors. This approach is of the utmost relevance to the *Insulin Resistance Phenotype* and the *Vascular Risk Phenotype* assessments since no effect indicators alone would be reasonable for measuring these phenotypes in adolescents. Factor loadings above 0.50 were adopted as criteria for evaluating convergent validity in Confirmatory Factor Analysis (CFA) for the latent variables which tested whether the theoretical factor structure was adequate for the observed data. Parameters of Root Mean Square Error of Approximation (RMSEA) were adopted with the upper limit of 90% confidence interval below 0.08 and CFI (Comparative Fit Index) and TLI (Tucker-Lewis Index) > 0.95. The Weighted Least Square Mean and Variance Adjusted (WLSMV) estimator and theta parameterization were used in the SEM to determine whether the data fitted the theoretical model, considering the same estimates described for CFA [15] using the software Mplus 8.0^®^.

## Results

The study included 2,515 adolescents aged between 18 and 19 years, 52.5% (n = 1,319) were female, 69.9% (n = 1,758) had completed high school, 50.2% (n = 1,116) belonged to socioeconomic class C, and 43.1% (n = 1,085) from a family with income of 1 and  < 2 monthly minimum wages. Regarding the household head´s education, 59.3% (n = 1,339) had completed high school. Harmful use of alcohol was identified in 19.4% (n = 489) of participants (Table 1). The mean fat mass index was 6.38 kg/m^2^ (SD ± 3.07), TG-HDL ratio was 2.05 (SD ± 1.72), TyG index was 8.22 (SD ± 0.46), VLDL concentration was 18.13 mg/dL (SD ± 9.43), SBP value was 113.91 mmHg (SD ± 12.13), DBP value was 70.69 mmHg (SD ± 7.30), and PWV  value was 5.50 m/s (SD ± 0.88).

**Table 1:** Socioeconomic characteristics and alcohol consumption of adolescents in São Luís, Brazil (2022)

Variable	n	%
Sex		
Male	1196	47.5
Female	1319	52.5
Adolescent’s education		
Elementary school	83	3.3
High school	1758	69.9
University education	672	26.8
Household head’s education		
Elementary School	593	26.3
High school	1339	59.3
University education	325	14.4
*CEB		
A	94	4.2
B	566	25.4
C	1116	50.2
D/E	450	20.2
Household income		
≤1 MW^†^	802	31.9
1–2 MW	1085	43.1
3–4 MW	341	13.6
≥5 MW	287	11.4
Alcohol		
Low risk	2026	80.6
High risk	489	19.4

^*^Brazil economic classification

^†^Minimum wage (R$ 880.00)

The male and female models showed reasonable fit rates (Table 2). All indicators of the latent variables *Family Socioeconomic Status*, *Insulin Resistance Phenotype* and *Vascular Risk Phenotype* showed good convergent validity with factor loadings above 0.5 for both sexes. VLDL was the indicator with the highest factor loading in males (standardized coefficient CP = 0.990; p < 0.001) and in females (CP = 0.969; p < 0.001), that accounted for almost all of the latent Resistance to Insulin (Table 3).

**Table 2:** Fit indices in the structural equation models for association between insulin resistance phenotype and vascular risk phenotype in adolescents. São Luís, Brazil (2022)

Model fit index	Expected index	Found index	Found index
		Male	Female
*X^2^		240.869	342.131
Degrees of freedom		132	132
*P* value *X*^*2*^		< 0.0001	< 0.0001
^†^RMSEA	<0.06	0.058	0.058
^‡^90% CI	< 0.08	0.054–0.063	0.054–0.063
C^§^FI	> 0.95	0.979	0.979
||TLI	> 0.95	0.974	0.974

^*^Chi-square test

^†^Root mean square error of approximation

^‡^Confidence interval

^§^|Comparative fit index

||Tucker Lewis index

**Table 3:** Factor loading, standard error and *p*-values of the latent variables indicators: family socioeconomic status, insulin resistance phenotype and vascular risk phenotype. São Luís, Brazil (2022)

Latent variable	Male			Female		
	Standardized coefficient	Standard error	*P*-value	Standardized coefficient	Standard error	*P*-value
*Family socioeconomic status						
Household head’s education	0.646	0.023	< 0.001	0.641	0.022	< 0.001
Socioeconomic class	0.853	0.025	< 0.001	0.894	0.022	< 0.001
Adolescent’s education	0.534	0.027	< 0.001	0.566	0.027	< 0.001
Monthly income	0.499	0.023	< 0.001	0.533	0.022	< 0.001
^†^Insulin resistance phenotype						
TG-HDL ratio	0.860	0.007	< 0.001	0.774	0.003	< 0.001
VLDL	0.990	0.006	< 0.001	0.969	0.003	< 0.001
TyG	0.940	0.011	< 0.001	0.800	0.005	< 0.001
^‡^Vascular risk phenotype						
SBP	0.737	0.016	< 0.001	0.825	0.015	< 0.001
DBP	0.672	0.015	< 0.001	0.723	0.012	< 0.001
PWV	0.530	0.015	< 0.001	0.618	0.016	< 0.001

^*^Family socioeconomic status: latent variable defined by the household head´s education, socioeconomic class according to CEB criteria, adolescent´s education, and monthly household income; ^†^*Insulin Resistance Phenotype*: defined by the Triglyceride to HDL ratio (TG-HDL), VLDL and Triglyceride Glycemic Index (TyG) ratio. ^‡^*Vascular Risk Phenotype*: Systolic Blood Pressure (SBP); Diastolic Blood Pressure (DBP), and Carotid-Femoral Pulse Wave Velocity (PWV)

The *Insulin Resistance Phenotype* was associated with the *Vascular Risk Phenotype* in males (CP = 0.183; p < 0.001) and in females (CP = 0.152; p < 0.001). In addition, the *Insulin Resistance Phenotype* was an indirect pathway for the association between harmful use of alcohol and the *Vascular Risk Phenotype* in males (CP = 0.020; p = 0.006) and in females (CP = 0.014; p = 0.039). The *Insulin Resistance Phenotype* was also an indirect pathway for the association of higher values of fat mass index and *Vascular Risk Phenotype* in males (CP = 0.054; p < 0.001) and in females (CP = 0.041; p < 0.001) (Fig. 2).

As secondary findings, higher values of Socioeconomic Status were associated with lower values of *Insulin Resistance Phenotype* in males (CP = −0.081; p = 0.028) and in females (CP = −0.126; p < 0.001). Family Socioeconomic Status was also associated with lower values of the *Vascular Risk Phenotype*, but only in females (CP = −0.157; p < 0.001), while higher values of Family Socioeconomic Status were associated with higher values of male fat mass index (CP = 0.229; p < 0.001). Harmful use of alcohol was associated with the *Insulin Resistance Phenotype* in males (CP = 0.112; p = 0.001) and in females (CP = 0.092; p = 0.036). Higher values of fat mass index were also associated with the *Insulin Resistance Phenotype* in males (CP = 0.296; p < 0.001) and in females (CP = 0.273; p < 0.001), and it was also directly associated with higher values of the *Vascular Risk Phenotype* in males (CP = 0.191; p < 0.001) and in females (CP = 0.296; p < 0.001) (Fig. 2).

## Discussion

Our findings add to the evidence that early events of continuums of diabetes and cardiovascular disease are already present in adolescents, showing an association between the *Insulin Resistance Phenotype* and the *Vascular Risk Phenotype* in both sexes. The *Insulin Resistance Phenotype* was also an indirect association pathway between higher values of fat mass index and the *Vascular Risk Phenotype*. Harmful use of alcohol was directly associated with the *Insulin Resistance Phenotype* and indirectly associated with the *Vascular Risk Phenotype* through the pathway of increased values of the *Insulin Resistance Phenotype*.

The *Insulin Resistance Phenotype* showed high convergent validity (standardized coefficient SC > 0.7) for the indicators TG-HDL ratio, TyG index and VLDL concentrations in both sexes. The increase in triglycerides is the crucial event in the alteration of insulin sensitivity, already observed in white adipose tissue [16] and the liver [6]. The TG-HDL ratio has been identified as an early marker of insulin resistance, capable of predicting Type 2 Diabetes [17]. Furthermore, the TG-HDL ratio is more efficient than HOMA-IR for diagnosing insulin resistance in obese young people [18]. The TyG index is also a marker of insulin resistance based on the increase in the flow of free fatty acids from adipose tissue to the liver and increase in hepatic triglycerides, which are strong determinants of hepatic insulin resistance [13]. Concurrently, hepatic synthesis of triglycerides results in increased production of VLDL cholesterol [7].

All indicators (SBP, DBP and PWV) of the latent variable *Vascular Risk Phenotype* had convergent validity for both sexes. These results confirm previous findings; however, using a population-based sample that was 3.85 times larger than our anterior study (n = 653) [11]. Advantageously, the *Vascular Risk Phenotype* represents incipient vascular alterations linked to vascular risk in young people while not requiring the adoption of a cut-off point to address vascular risk in this population.

Higher values of fat mass index were directly associated with the *Insulin Resistance Phenotype*. This phenotype was an indirect pathway in the association between higher values of fat mass index with the *Vascular Risk Phenotype*. The increase in adipocytes results in hypoxia and consequently the accumulation of macrophages in adipose tissue, which is the primary source of inflammatory mediators that can induce insulin resistance [19]. Oxidative stress in obesity can culminate in mitochondrial dysfunction and consequently lipid accumulation, accelerating both insulin resistance [20] and atherosclerosis [21]. Insulin resistance has been explained from the lipocentric view. The collection of intramuscular lipids from the entry of fatty acids into cells inhibits the translocation of GLUT-4 to the plasma membrane, leading to the loss of insulin-dependent glucose uptake [22].

Higher values of fat mass index were also directly associated with the *Vascular Risk Phenotype*, which could be due to excess fat, leading to increased blood pressure via elevated sympathetic activity, subsequent sodium reabsorption, and peripheral vascular resistance [23].

The *Insulin Resistance Phenotype* and the *Vascular Risk Phenotype* were also directly associated. As a possible explanation, excessive dietary carbohydrate intake induces insulin resistance in skeletal muscle, which can promote atherogenic dyslipidemia by diverting carbohydrates used in muscle glycogen storage to de novo* lipogenesis* (Fig. 2), resulting in elevated plasma triglyceride, decreased HDL, elevated small and dense LDL and reduced hepatic triglyceride synthesis, and consequently, increasing VLDL [24]. Added sugars activate hepatic lipogenesis pathways from glucose [25] and fructose [26] by increasing plasma triglyceride levels and result in dyslipidemia [27], insulin resistance [28] and type 2 diabetes [29] regardless of fat. Fructose induces the faster formation of fatty acids compared to glucose since it lacks hepatic feedback regulatory mechanisms [30], increasing de novo* lipogenesis* and liver fat, decreasing insulin sensitivity, regardless of weight gain [31], contributing to the greater secretion of VLDL [32].

VLDL was the indicator of the *Insulin Resistance Phenotype* showing the highest factor loading in both sexes, emerging in our study as an insulin resistance marker linked to cardiovascular risk in young people. Research using VLDL as a biological marker is scarcer, even though VLDL is associated with insulin resistance [14] and CVD [33]. Elevated plasma triglyceride and VLDL determine greater insulin secretion in obese adolescents, regardless of initial insulin sensitivity [14]. Insulin resistance plays an essential role in VLDL metabolism by increasing the availability of apolipoprotein B (ApoB), the primary lipoprotein of VLDL, leading to the increased hepatic synthesis of VLDL [34]. At the same time, VLDL concentration is essential in the initiation and progression of atherosclerosis [33] and can manifest in young people [35].

Harmful use of alcohol was directly associated with the *Insulin Resistance Phenotype*. In addition, the harmful use of alcohol in young people was indirectly associated with the *Vascular Risk Phenotype* through increased *Insulin Resistance Phenotype* in both sexes. Excessive alcohol consumption is linked to oxidative stress, inflammatory responses [36] and hypertension [37].

More distally, higher values of *Family Socioeconomic Status* were associated with lower values of the *Insulin Resistance Phenotype* in both sexes. Higher values of *Family Socioeconomic Status* were also associated with lower values of the *Vascular Risk Phenotype*, but only in females, confirming a previous study that shows the highest cardiovascular risk and incredibly high disease burden for low-income groups [38]. Low *socioeconomic status* groups have a higher risk of developing cardiovascular disease [39]. People with lower socioeconomic status may be more vulnerable to CVD and diabetes due to material deprivation, unsanitary living conditions, limited access to high-quality health care, reduced opportunities to prevent complications, inappropriate, risky behavior such as tobacco use, unhealthy foods habits, a sedentary lifestyle and being overweight or obese [40]. A possible explanation for this difference between the sexes in our study is that higher values of *Family Socioeconomic Statu*s were associated with higher values of fat mass index in boys. In addition, cardiovascular risk is also higher for males [41], which could be explained by hormonal differences, possibly linked to estrogen activities or factors associated with the X chromosome that protect vascular walls in females [42].

The main limitation is that the cross-sectional design did not allow us to assume a temporal link among the exploratory variables, Insulin Resistance Phenotype, and the vascular outcome. Furthermore, we cannot rule out reverse causality in shown associations, for example, the inverse pathway of the Insulin Resistance Phenotype increasing the fat mass index. Some bias may arise from losing participants from the original Cohort from birth and the necessity to open the sample at 18–19 follow-up to survey representativeness. In this respect, to guarantee the sample power fellow was opened using official Brazilian data (SINASC). In this respect, we compared some characteristics between the original cohort and new participants from SINASC (retrospective cohort) related to head of the family, economic class, monthly family income, alcohol consumption, and fat mass index; however, we did not find statistically significant differences between these populations.

The study’s strengths lie in the construction of the latent variable *Insulin Resistance Phenotype* aimed at reducing the measurement error of early markers of insulin resistance and test the validity of the *Vascular Risk Phenotype* in a population sample stratified by sex to estimate incipient vascular changes in adolescents.

## Conclusions

The contribution of this study has been to estimate the association between early events of diabetes and cardiovascular continuums through both the *Insulin Resistance Phenotype* and *Vascular Risk Phenotype*. Lower values of socioeconomic status, harmful use of alcohol, and higher values of body fat were also associated with the two phenotypes. The association of *Insulin Resistance Phenotype* with *Vascular Risk Phenotype* in young people suggests common pathophysiological mechanisms in diabetes and cardiovascular continuums.

## Data Availability

The datasets used and analyzed during the current study are available from the corresponding author on reasonable request.

## Abbreviations

MACE: Major adverse cardiac event
CVD: Cardiovascular diseases
VLDL: Very-Low-Density lipoprotein
TG/HDL: Triglyceride to HDL ratio
TyG: Triglyceride glycemic index
SBP: Systolic blood pressure
DBP: Diastolic blood pressure
PWV: Carotid-Femoral pulse wave velocity
SINASC: Brazilian live birth information system
AUDIT: Alcohol use disorders identification test
DEXA: Dual-energy x-ray absorptiometry
TG: Triglycerides
HDL: High-Density lipoprotein
SEM: Structural equation modeling
CFA: Confirmatory factor analysis
RMSEA: Root mean square error of approximation
CFI: Comparative fit index
TLI: Tucker-Lewis index
WLSMV: Weighted least square mean and variance adjusted
SC: Standardized coefficient
